# The evolution of mesenchymal stem cell-derived neural progenitor therapy for Multiple Sclerosis: from concept to clinic

**DOI:** 10.3389/fncel.2024.1428652

**Published:** 2024-08-29

**Authors:** Majid Ghareghani, Ayanna Arneaud, Serge Rivest

**Affiliations:** Neuroscience Laboratory, CHU de Québec Research Centre, Department of Molecular Medicine, Faculty of Medicine, Laval University, Québec City, QC, Canada

**Keywords:** Multiple Sclerosis, mesenchymal stem cell-derived neural progenitors, regenerative therapy, clinical trials, neuroimmunology

## Abstract

This review delves into the generation and therapeutic applications of mesenchymal stem cell-derived neural progenitors (MSC-NPs) in Multiple Sclerosis (MS), a chronic autoimmune disease characterized by demyelination, neuroinflammation, and progressive neurological dysfunction. Most current treatment paradigms primarily aimed at regulating the immune response show little success against the neurodegenerative aspect of MS. This calls for new therapies that would play a role in neurodegeneration and functional recovery of the central nervous system (CNS). While utilizing MSC was found to be a promising approach in MS therapy, the initiation of MSC-NPs therapy is an innovation that introduces a new perspective, a dual-action plan, that targets both the immune and neurodegenerative mechanisms of MS. The first preclinical studies using animal models of the disease showed that MSC-NPs could migrate to damaged sites, support remyelination, and possess immunomodulatory properties, thus, providing a solid basis for their human application. Based on pilot feasibility studies and phase I clinical trials, this review covers the transition from preclinical to clinical phases, where intrathecally administered autologous MSC-NPs has shown great hope in treating patients with progressive MS by providing safety, tolerability, and preliminary efficacy. This review, after addressing the role of MSCs in MS and its animal model of experimental autoimmune encephalomyelitis (EAE), highlights the significance of the MSC-NP therapy by organizing its advancement processes from experimental models to clinical translation in MS treatment. It points out the continuing obstacles, which require more studies to improve therapeutic protocols, uncovers the mechanisms of action, and establishes long-term efficacy and safety in larger controlled trials.

## Introduction

Multiple Sclerosis (MS) is an autoimmune debilitating disease, which progressively damages the central nervous system (CNS). The outcome is a variety of physical and cognitive problems. Demyelination, inflammation, and neurodegeneration are the central features of MS, which disrupts neural communication and leads to a multitude of symptoms, from mild numbness to severe paralysis and visual impairment. A large part of conventional therapies are focused on the immune response; thus, they fail to work in the neurodegenerative aspect of the disease.

MS and its animal model experimental autoimmune encephalomyelitis (EAE) have distinct molecular and cellular processes that define disease course and treatment outcome. In the initial stages of MS and EAE, autoreactive T cells are activated against the specific antigens of the CNS, which in turn cause the proliferation of the pathogenic T cells and their infiltration into the CNS resulting in inflammation and demyelination. These immune cells release cytokines and chemokines that in turn attract and activate more immune cells, thus creating a vicious cycle of neuroinflammation. In the later stages of the disease, the condition becomes chronic, characterized by constant inflammation that results in the deterioration of neurons and the buildup of disability (Robinson et al., 2014). Microglia, astrocytes and peripheral immune cells are the major players in inflammation as they not only have high potential for amplify the inflammatory response but also mediate neuronal damage via oxidative stress and excitotoxicity (Li et al., 2022). The new era of regenerative medicine progressions has brought in mesenchymal stem cell-derived neural progenitors (MSC-NPs) as a radical therapeutic concept that aim to modulate the immune response, reduce inflammation, and support remyelination by differentiating into oligodendrocyte progenitor cells and secreting neurotrophic factors. Understanding these phases and cellular interactions is crucial for developing effective treatments and tailoring interventions to different stages of the disease.

This review begins by addressing the studies conducted on mesenchymal stem cells (MSC) at both the experimental level using the EAE model of MS and in MS patients. It then discusses the developmental process of MSC-NP therapy which involves the emergence of the concept, experimental evidence, and clinical application. In the initial stage, the therapeutic effect of MSC-NPs was tested at the experimental level by using both *in vitro* models and animal models of MS, in which their ability to migrate to the sites of injury was assessed, the remyelination was promoted, and the inflammatory environment of the CNS was affected. The positive outcomes from the preclinical studies, in fact, shaped the foundation for the advancement to clinical trials. As a result, this sheds light on the well-tolerated nature, feasibility, and efficacy of the MSC-NP therapy in humans.

## MSCs therapeutic strategies in EAE models and MS patients

Research has progressively elucidated the role of MSC in modulating immune responses within the context of MS and its animal model, EAE. Beginning with pivotal research by Freedman et al. in 2005, which demonstrated the therapeutic and preventive potential of MSCs in EAE, the groundwork was laid for understanding MSCs' modulatory functions. This study demonstrated that the intravenous administration of MSC before or at different stages of EAE, but not after disease stabilization, prevented symptoms, CNS inflammation, demyelination as well as T-cell/macrophage infiltration. These findings represent both therapeutic and preventive effects. A crucial finding demonstrated that MSC treatment caused T-cell anergy, which could be restored by IL-2. This implies that the immunomodulatory effects are the mechanism of action rather than direct neural regeneration. This conclusion was further confirmed in the study by Gerdoni et al. conducted in 2007 which found that the EAE mice treated with green fluorescent protein (GFP) tagged MSCs developed less severe symptoms, fewer relapses, and less CNS inflammation without any signs of MSC differentiation into neural cells. This investigation identified a function of MSCs in modulation of autoimmune phenomena, including induction of T-cell anergy and decreasing PLP-specific T-cell responses (Gerdoni et al., 2007). However, in 2008, Kassis et al. opposed the earlier study by Gerdoni et al. (2007), by reporting that intraventricularly injected GFP-tagged MSCs can differentiate into neuronal and glial lineage cells, thus, such cells can have the potential for direct neural regeneration and remyelination. This approach demonstrated a marked improvement in clinical symptoms and neurodegeneration in chronic EAE models (Kassis et al., 2008).

Subsequent studies have continued to explore MSCs' dual roles in immunomodulation and neurodegeneration. For instance, Lu et al. investigated the MSC improvement with ciliary neurotrophic factor (CNTF) for remyelination and functional recovery in mice with EAE. This approach resulted in significant clinical and immunological improvements, a decrease in the levels of inflammatory cytokines, and a rise of NG2-positive oligodendrocyte precursor cells, indicating a dual approach of immunomodulation and myelin repair (Lu et al., 2009). In the subsequent year, the report by Rafei et al. employed allogeneic MSCs from BALB/c donors to treat EAE in C57BL/6 mice and assess the viability of “universal donor” MSCs in autoimmune therapy. The outcomes showed a notable reduction of EAE severity, spinal cord immune cell infiltration, and levels of the pro-inflammatory cytokines IFN-γ and IL-17, which was comparable to the effects with syngeneic MSCs. However, the study also observed a potential limitation of allogeneic MSCs: a decline in the suppressive activity following IFN-γ pretreatment associated with increased CCL2, MHC I, and MHC II expression and allogeneic rejection. This highlights the complexity of MSC-mediated immunomodulation, and, hence, a prudent use of allogeneic MSCs as a treatment choice because of potential immunogenicity and alloimmunization risks. Nonetheless, the results of the research demonstrate the effect of MSC-derived CCL2 immunosuppression, which reflects the possibility of MSCs as a treatment for autoimmune diseases like MS (Rafei et al., 2009). Additionally, Constantin et al. (2009) examined the therapeutic potential of adipose-derived MSCs (aMSCs) as the preventive and therapeutic option for EAE. Pre-onset intravenous aMSC treatment attenuated the severity of EAE through an immune modulation that featured reduction in spinal cord inflammation, demyelination, axonal loss, and a Th2 type cytokine pattern. aMSCs exhibited dual homing in two places i.e., lymphoid organs and CNS, where some were α4 integrin-expressing activated for attachment to inflamed venules of the brain, perhaps having both early immune response suppression and late-stage neuroregeneration benefits. Nevertheless, aMSC treatment enhanced the population of the endogenous oligodendrocyte progenitors in the demyelinating region. The given dual mechanism represents the diversified therapeutic actions of aMSCs in autoimmune neurodegenerative disorders (Constantin et al., 2009).

The journey from experimental models to clinical application has seen MSCs' capabilities being harnessed more effectively. In a notable 2010 study by Gordon et al., human MSCs (hMSCs) administered intravenously were shown to reduce the severity of EAE in mice. It should be noted that two morphologies of hMSCs that were found in the CNS in this study suggest that these cells have different functions or states upon infiltration. Some cells expressed neural markers and were linked to low demyelination, implying neuroprotective or reparative functions (Gordon et al., 2010). The following year, in 2011, Grigoriadis introduced an intracerebroventricular (ICV) type of transplantation of bone MSCs on EAE models, showing the remarkable improvement of mild EAE symptoms with anti-inflammatory effects on the spinal cord and axonopathy reduction. This investigation was dedicated to the migration of MSCs to the brain producing cellular deposits in parts of intense EAE characterized by inflammation, demyelination, axonal loss, and increased extracellular matrix deposition via upregulation of connective tissue growth factor (CTGF) and likely induced by TGFβ1 (Grigoriadis et al., 2011). Further expanding on this theme, Yousefi et al. (2013) study showed that intravenous and intraperitoneal administration of aMSCs in EAE were both effective in decreasing clinical severity for either method. Notably, the intraperitoneal approach was more effective in the modulation of immune responses as demonstrated by increased populations of regulatory T cells and decreased IL-4. The increase in the CD4^+^CD25^+^FOXP3^+^ T cell population in the spleen was more significant in the intraperitoneal-administered group, suggesting a stronger systemic immunomodulatory effect through this route (Yousefi et al., 2013).

A study by Dang et al. revealed that autophagy is induced in MSCs within the inflammatory environment of EAE mice via cytokines such as IFN-γ and TNF. This mechanism was accomplished through either BECN1 knockdown or pharmacological treatment with 3-methyladenine (3-MA). MSCs autophagy repression led to the increased therapeutic potential of these cells reflected in reduced CD4+ T cell activation and proliferation, and, therefore, autophagy modification in MSCs was proposed as another approach to amplify the immunosuppressive and therapeutic effects in autoimmune diseases (Dang et al., 2014). By 2015, the understanding of MSCs had evolved to recognize their nuanced roles in immune modulation and neuroprotection. In 2015, Kurte and colleagues reported that MSCs could improve EAE symptoms in a classic manner or induce atypical symptoms such as unbalanced gait or rotatory defects, especially when administered at peak or post-stabilization of the disease. This effect did not depend on Th17/Th1 ratios, implying different modes of action. In the EAE brain, treatment significantly decreased pro-inflammatory markers (IL-6, T-bet, and RORγT) and increased regulatory markers (Foxp3), suggesting a wide immunomodulatory effect and restoration of blood-brain barrier (BBB) function (Kurte et al., 2015).

A study by Glenn et al. (2015) noted that MSC intervention during the EAE priming stage did not affect the disease severity, though it did inhibit TH17 cell proliferation and differentiation *in vitro*, without changing TH1 cells. Although, MSCs led to increased IFNγ production by TH1 cells *in vitro* and *in vivo*, thereby raising a possibility of a TH1-mediated response (Glenn et al., 2015). Liao et al. (2016) modified MSCs to produce P-selectin glycoprotein ligand-1 (PSGL-1), Sialyl-Lewis^∧^x (SLeX), and interleukin-10 (IL-10), which improved their therapeutic effect in EAE. This engineering drastically enhanced MSC homing to the inflamed spinal cord of EAE mice, this was achieved through increased rolling and adhesion on activated brain microvascular endothelial cells, which are part of the inflamed endothelium of the blood-brain/spinal cord barrier in EAE. Furthermore, MSCs transfected with IL-10 mRNA exhibited enhanced inhibition of CD4^+^ T cell proliferation *in vitro*, highlighting their potent immunosuppressive function (Liao et al., 2016).

Similarly, Bravo et al. (2016) used decidua-derived MSCs (DMSCs) in EAE and reported that IP injections significantly delayed the onset of symptoms and that, when given after moderate symptoms had appeared, led to a milder disease course. It has been attributed to reduced inflammatory invasion in the CNS and a change from a Th17 to a Th2 immune response, which underlines DMSC's ability to suppress Th17 immune pathways (Bravo et al., 2016).

Continuing this line of research, in the study by Jiang et al. (2017), the effects of placental-derived MSCs (PMSCs) and embryonic MSCs (EMSCs) were compared in the EAE model, and both were found to be effective in the amelioration of EAE (Jiang et al., 2017). This comparison was further investigated by Singh et al., between multipotent adult progenitor cells (MAPCs) and MSCs, with MAPCs showing better treatment outcomes in EAE, implying diverse abilities in different types of stem cells in autoimmune therapy (Singh et al., 2017).

The therapeutic potential of nanovesicles (NVs) from aMSCs in treating EAE and substantially reversing its symptoms were emphasized by Farinazzo et al. (2018). The aMSCs derived NVs partially alleviated the severity of the disease and modulated the immune response with only mild effects on T cell proliferation but rather their action might be in suppressing microglial activation and T cell infiltration into the CNS. Indeed, aMSCs-NVs were found to inhibit both basal and LPS-induced proliferation of the N9 murine microglial cell line *in vitro*, as well as reduce the number of activated microglial cells in the spinal cord of EAE mice. It also inhibited integrin-dependent adhesion of activated T cells to ICAM-1 and VCAM-1 *in vitro*, especially when adhesion was triggered by the chemokine CXCL12. This effect was not due to reduced expression of adhesion molecules by T cells, indicating that aMSC-NVs interfere with the signaling required for integrin activation by chemokines (Farinazzo et al., 2018). Jafarinia et al. further supported the success of MSC-derived factors in EAE treatment by showing the beneficial effects of extracellular vesicles of human aMSCs, thereby reinforcing the therapeutic potential of MSC-based interventions (Jafarinia et al., 2020).

Recent studies have continued to build on these findings. Zhang et al. (2021) demonstrated bone marrow-derived MSCs' involvement in promoting the oligodendrocyte lineage in EAE, as evidenced by augmented numbers of progenitor and mature oligodendrocytes. This is indicative of both direct support for remyelination and neural repair (Zhang et al., 2021). Manganeli Polonio et al. (2021) showed that treatment of endometrial-derived MSCs (meMSCs) in EAE led to a reduction of Th1 and Th17 lymphocytes which were related to the improvement of anti-inflammatory markers such as IL27 and IL-10-cytokine-secreting T cells. The study revealed that Indoleamine-2,3-Dioxygenase (IDO) played a crucial role in the therapeutic mechanism, demonstrating that the IDO-kynurenines-AhR axis is important in MSC-mediated immune modulation (Manganeli Polonio et al., 2021).

More recently, Wang et al. (2023) found that BM-MSCs-Exosomes with miR-23b-3p greatly decreased microglial pyroptosis by targeting NEK7, involving a new pathway in MSC therapy's mechanism against neuroinflammation (Wang et al., 2023). A similar study by Haghmorad et al. concerning the alteration of miRNA expression by MSCs in EAE provided new indications about the molecular mechanisms behind efficacy of MSC therapy. This emphasized the regulatory effect of certain miRNAs on immune responses and inflammation including miR-193, miR-146a, miR-155, miR-21, and miR-326 (Haghmorad et al., 2023).

The promising results from experimental models facilitated the transition of MSC therapies into clinical trials aimed at evaluating their potential in treating human MS. Initial clinical applications, as demonstrated by Yamout et al. in 2010, evaluated the risk profile and therapeutic potential of intrathecal injection of autologous BM-MSCs in 10 MS patients. In the case of patients who were treated 3–6 months post-treatment, five out of seven patients improved according to the Expanded Disability Status Scale (EDSS) scores, one score remained the same, and one score worsened by 0.5 points. In three patients, these changes were sustained and in three others, they were stabilized at the 12-month follow-up. Even with clinical improvements, MRI data at 3 months revealed new or enlarging lesions in five out of seven patients and Gadolinium-enhancing lesions in three. Magnetic Resonance Spectroscopy (MRS) showed a decrease in N-acetylaspartate/Creatine (NAA/Cr) ratio, indicating continuing neuronal damage despite the treatment (Yamout et al., 2010).

The same year, the transplantation of MSCs in 15 patients with MS was well-tolerated and offered potential clinical and immunological advantages. Functional improvement was observed in patients with MS. Another set of data regarding immunological changes post-transplantation indicates a down-regulation of immune responses that might be responsible for the clinical benefits (Karussis et al., 2010). An open label study and a pilot clinical trial on 25 and 26 progressive MS patients, respectively, confirm that autologous MSC therapy can improve/stabilize the course of the disease with no serious adverse effects (Bonab et al., 2012; Cohen et al., 2018). Another clinical study by Mohajeri et al., was dedicated to evaluating FoxP3 expression, a specific marker of T regulatory cells, in 7 MS patients, before and after MSC therapy. The upregulation of FoxP3 in peripheral blood mononuclear cells was found to be more pronounced 6 months after intrathecal injection of MSC in almost all subjects, indicating an increased regulatory T cell activity that could play a role in the clinical stability of MS patients (Mohajeri et al., 2011).

Another clinical study on 24 progressive MS patients reported that repeated intrathecal and intravenous administrations of autologous MSCs were safe over the course of 4 years with no severe treatment-related adverse events. Most patients showed clinical improvements or stability, particularly those receiving more than two treatments, and a statistically significant reduction in the mean EDSS score was observed from baseline to the last visit. Immunological evaluations demonstrated a transient rise in regulatory T cells and a reduction in lymphocyte proliferation, indicating short-term immunomodulatory effects (Petrou et al., 2021). Another study reported the CSF biomarkers of 48 progressive MS patients after MSCs, such as neurofilament light chains (NFL) and the chemokine receptor CXCL13. NFL levels, a biomarker of neurodegeneration, decreased significantly 6 months post-IT MSC treatment when compared to baseline, indicating a possible neuroprotective effect. This reduction was significantly greater in the intrathecal treatment group than in the intravenous treatment and placebo groups. On the contrary, the levels of CXCL13, markers of CNS inflammatory activity, tended to decrease in the MSC-intrathecal group compared to the placebo, but the difference was not significant (Petrou et al., 2022).

In a pilot trial evaluating the effectiveness of umbilical cord MSCs (UCMSC) transplantation for MS treatment, two cases of UCMSC treated patients demonstrated ameliorated symptoms, decreased clinical attacks, and improvements in MRI and neurological function scores. The adverse reactions were usually mild and passing. The UCMSCs showed a capacity for differentiation into different cell types and had normal karyotypes, which indicated their safety for transplantation. Significantly, this therapy regulated the immune response of patients, demonstrated through reduced mRNA expression of CD86, IL-2, CTLA-4, and HLADRB1 in peripheral blood after transplantation (Meng et al., 2018).

Another clinical trial of 25 MS patients receiving UCMSC transplantation demonstrated the capability of the cells to differentiate into different cell types and expressed UCMSC markers. The adverse reactions were mild and transient with fever, dizziness, and headache. Post-transplant, improvements were noted in symptoms, vital signs, neurological function scores, and MRI findings indicating decreased disease activity. An immunomodulatory effect of UCMSC transplantation was also noted by the decrease in pro-inflammatory cytokines and an increase in regulatory T cells. Nevertheless, significant differences were found in regards to the rate of cell growth and dose achievement in the participants with no correlations between the growth rates and patient demographics or disease characteristics (Planchon et al., 2018).

In evaluating the outcomes of allogeneic vs. autologous MSC therapy in EAE models, both types have shown beneficial effects. However, no direct comparisons between these two sources have been extensively reported. Notably, allogeneic MSCs, such as those isolated from C57BL/6J mice and injected into SJL/J mice, do not typically transdifferentiate into neural cells, despite migrating to the CNS (Gerdoni et al., 2007). In contrast, autologous MSCs, derived from and injected back into the same genetic background (e.g., C57BL6 mice), demonstrate a higher potential for differentiation into neural and glial cells within the CNS (Kassis et al., 2008). This suggests that while both types of MSCs are effective in modulating immune responses in EAE, autologous MSCs may offer advantages in terms of cellular compatibility and minimizing the risk of immune rejection.

In summary, the findings from both experimental and clinical studies provide evidence on the therapeutic role of MSCs in the context of MS treatment. They uncover their ability to control immune reactions, increase neuroprotection, and result in clinical improvement or stabilization. However, further studies should be done to determine the mechanisms, improve the treatment protocols, and determine the long-term efficacy and safety of MSC therapy in larger controlled trials. The routes of the injections, the source of MSCs, and the key findings from studies on the EAE model are summarized in Table 1.

**Table 1:** Studies on the role of MSCs in EAE model and key findings.

**References**	**Injection**	**Type of cell**	**Key findings**
Gerdoni et al. (2007)	Intravenous	allogeneic GFP-tagged MSCs	Reduced PLP-specific T-cell responses, T-cell anergy
Kassis et al. (2008)	Intraventricularly and/or intravenously	autologous GFP-tagged MSCs	Differentiation into neuronal and glial lineage cells
Lu et al. (2009)	Intravenous	Human MSCs enhanced with CNTF	Reduced inflammatory cytokines, INCREASED NG2-positive cells
Rafei et al. (2009)	Intraperitoneal	Allogeneic MSCs	Reduced IFN-γ, IL-17, increased CCL2 (after IFN-γ pretreatment)
Constantin et al. (2009)	Intravenous	Adipose-derived MSCs (aMSCs)	Reduced spinal cord inflammation, increased oligodendrocyte progenitors
Gordon et al. (2010)	Intravenous	Human MSCs (hMSCs)	Distinct cell morphologies, Some expressing neural markers, hMSCs accumulation over time in demyelinated areas
Grigoriadis et al. (2011)	Intravenous intrathecal	autologous MSCs	reduced axonopathy only in the mild EAE, Increased CTGF under the trigger of TGFb1, formed cellular masses
Yousefi et al. (2013)	Intraperitoneal intravenous	allogeneic aMSCs	Increased regulatory T cells, Decreased IFN-γ
Dang et al. (2014)	Intravenous	MSCs	Inhibition of autophagy enhances therapeutic effects
Kurte et al. (2015)	Intravenous	MSCs	Decreased IL-6, T-bet, RORγT, Foxp3 mRNA
Glenn et al. (2015)	Intraperitoneal	MSCs	Increased IFNγ by TH1 cells, No significant effect on TH1 cells
Liao et al. (2016)	Intravenous	Engineered MSCs (PSGL-1, SLeX, IL-10)	Enhanced homing to inflamed spinal cord; enhanced therapeutic efficacy compared to unaltered MSCs
Bravo et al. (2016)	Intraperitoneal	Decidua-derived MSCs (DMSCs)	Decreased CD4(+)IL17(+), Shift from Th17 to Th2 response
Jiang et al. (2017) and Singh et al. (2017)	Intracerebroventricular	PKH26-labeled multipotent adult progenitor cells (mMAPCs) vs. MSCs. Placental-derived MSCs (PMSCs) vs. Embryonic MSCs (EMSCs)	Greater effectiveness in MAPCs compared to MSCs and PMSCs compared to EMSCs
Farinazzo et al. (2018) and Jafarinia et al. (2020)	Intravenous	Nanovesicles from aMSCs and Extracellular vesicles from human aMSCs	Inhibition of microglial activation and T cell infiltration
Zhang et al. (2021)	Intracerebroventricular	MSCs	Increased oligodendrocyte lineage numbers
Manganeli Polonio et al. (2021)		Endometrial-derived MSCs (meMSCs)	Increased Il27, IL-10-secreting T cells, upregulated IDO
Wang et al. (2023)	Intrathecal	MSC-Exosomes containing miR-23b-3p	Reduced microglial pyroptosis, targeted NEK7

## Therapeutic potential of MSC-NPs in experimental model of MS

In 2011, Sadiq's team embarked on a groundbreaking investigation to enhance myelination in shiverer mice, a model for dysmyelination in the CNS due to a genetic mutation impairing myelin sheath formation due to absence of myelin basic protein (MBP) production (Cristofanilli et al., 2011). Their innovative approach involved a double stem cell application strategy; embryonic stem cell (ESC)-derived oligodendrocyte progenitor cells (OPCs; allogeneic to the recipient) were transplanted alongside syngeneic MSCs directly into the adult immunocompetent shiverer mouse corpus callosum. This dual-transplantation strategy was based on the premise that MSCs could enhance the survival, migratory capability, and myelinating efficiency of the ESC-derived OPCs within the dysmyelinated CNS. The results were significant, showing that MSCs synergistically enabled a prolonged engraftment of allogeneic OPCs and a marked increase in myelination.

The MSCs played a crucial role in modulating the immune environment within the CNS, primarily by reducing the host's immune response against the allogeneic OPCs through the production of TGF-β. This activity minimized microglia activation and astrocytosis, thus preserving OPCs from immune-mediated rejection or damage and supporting their survival and integration into the host CNS (Cristofanilli et al., 2011).

Beyond their immunomodulatory functions, MSCs were essential in providing trophic support for the transplanted OPCs. The study highlighted the secretion of Insulin-like Growth Factor 1 (IGF-1) by MSCs, a critical factor in promoting OPC differentiation into myelinating oligodendrocytes. This trophic support not only expedited the differentiation of OPCs but also enhanced their capacity to form functional myelin sheaths around axons, normalizing the CNS function in the dysmyelinated shiverer mice. This study lays the groundwork for the potential of MSCs to improve the efficacy of allogeneic OPC transplantation in dysmyelinated conditions.

The following year, Sadiq's team shifted focus to the EAE mouse model, an established model for studying MS, in order to explore the reparative capabilities of MSC-NPs in a context closely mirroring the pathological conditions of MS (Harris et al., 2012a). They began by deriving and extensively characterizing a homologous MSC-NPs population from mice. The MSCs, isolated from C57BL/6 mice bone marrow, exhibited the typical adherent morphology and the characteristic MSC surface phenotype of CD9^+^/CD44^+^/Sca1^+^/CD45^−^. Using a protocol parallel to that used for human MSCs, the mouse MSCs were differentiated into MSC-NPs, which displayed a distinct neurosphere morphology indicative of their neural progenitor status.

A crucial step was the full characterization of their neural lineage potential. Gene expression analyses showed significant upregulation of key neural markers: Nestin (a neural stem cell marker), medium neurofilament (NFM, a neuronal marker), and GFAP (a marker for glial cells and stem cells). Notably, NFM expression surged, indicating a shift toward a neural progenitor-like profile. This transition was underscored by diminished expression of the MSC marker SMA and a slight decrease in Sca1, aligning with the surface expression profile of MSC markers in MSC-NPs (Harris et al., 2012a).

The multipotentiality test showed that while MSC-NPs expressed MSC markers, they lost most of their adipogenic and osteogenic potential when compared to MSCs. Hence, reduced mesodermal plasticity along with increased neural markers and decreased MSC markers indicated that the multipotent MSCs were transforming into a more lineage specific neural progenitor like cell population (Harris et al., 2012a).

To further explore their capabilities, the immunomodulatory properties of MSC-NPs were then investigated due to the ability of MSCs in suppressing the activation and proliferation of T cells. The co-culture experiments with the T cells activated by anti-CD3/CD28 showed that both MSC-NPs and MSCs suppressed the CD4+ T cell proliferation effectively. This suggests that MSC-NPs have the ability to modulate the immune response as it is known that MSCs play a role in the autoimmune component of MS (Harris et al., 2012a).

To directly evaluate the therapeutic efficacy of MSC-NPs in the EAE model, syngeneic MSC-NPs were intrathecally administered to mice with EAE that was induced by immunization with a myelin oligodendrocyte glycoprotein (MOG) peptide. The MSC-NPs were labeled with DiI, a fluorescent dye, prior to injection, in order to monitor their fate post transplantation. The pathway started at the beginning of the chronic phase as it was a better reflection of intervention in the established MS. Following the injection, the DiI-labeled MSC-NPs were found to spread throughout the CNS, with the focal point on regions attacked by autoimmune assault and demyelination, two of the hallmarks of the EAE model, closely replicating the pathological environment of MS (Harris et al., 2012a).

Histopathological analyses indicated a significant reduction in CD3+ T cell infiltration in areas containing DiI-labeled MSC-NPs, suggesting that MSC-NPs could modulate the local immune response. This modulation is likely achieved via the production of an anti-inflammatory milieu that suppresses the activation and proliferation of pathogenic T cells. The precise mechanisms underlying this immunomodulatory effect remain a focus of ongoing research but are thought to involve the secretion of anti-inflammatory cytokines and growth factors, contributing to immunologic homeostasis restoration and tissue healing. Additionally, regions with MSC-NP presence showed marked reductions in demyelination, as confirmed by MBP immunostaining. This indicates that MSC-NPs not only halt myelin damage progression but also contribute to the remyelination process, either directly by differentiating into myelinating cells or indirectly by supporting the survival and function of endogenous OPCs. Notably, the therapy appeared to enhance endogenous neural progenitors, as indicated by an increase in Nestin-positive cells in areas of DiI-labeled MSC-NP distribution, suggesting that MSC-NPs might strengthen the CNS's intrinsic repair mechanisms, possibly through trophic support that encourages the proliferation and differentiation of resident progenitor cells into myelinating oligodendrocytes or other neural cells. Furthermore, administering multiple doses of MSC-NP treatment resulted in a dose-dependent improvement in EAE scores over time (Harris et al., 2012a).

In the same year, Sadiq's team conducted another study aimed to evaluate the properties of MSCs derived from MS patients, and differentiate them into MSC-NPs, and use them as the therapeutic potential of these cells. This study played a crucial role in validating the use of MSCs from MS patients for therapeutic purposes, addressing concerns about the phenotypic normalcy and functional capabilities of these cells compared to those from healthy controls (Harris et al., 2012b). Their study began by characterizing MSCs isolated from MS patients to determine whether they met the International Society for Cell Therapy's minimal criteria for defining MSCs as multipotent. Both MS patient and healthy control derived MSCs exhibited similar growth kinetics, typical MSC morphology, and standard surface antigen expression, indicating that MS-MSCs were phenotypically normal and comparable to control MSCs.

Additionally, the study examined the neural differentiation ability of MSC-NPs derived from the MSCs derived from MS patient. MSC-NPs displayed neurosphere morphology and higher levels of neural markers of Nestin, NFM, GFAP, and CXCR4, while showing decreased expression of MSC markers such as CD90 and SMA. This differentiation to MSC-NP indicated the expression of progenitor-like markers, supported either by gene expression analysis or morphological changes. Unlike MSCs, MSC-NPs exhibited limited adipogenic and osteogenic differentiation capacity, highlighting a lineage-restricted differentiation potential. This characteristic is particularly important for CNS transplantation, as it mitigates the risk of unwanted mesodermal differentiation, ensuring safer application. The immunoregulatory properties of MSC-NPs were also studied, showing that both MSC-NPs and MSCs could suppress T cell proliferation and promote regulatory T cell generation. This retains the immunomodulatory characteristics of MSCs, which is vital for therapeutic applications, especially in autoimmune diseases like MS, where immune modulation is essential for treatment efficacy (Harris et al., 2012b).

Years later, in 2023, Sadiq's team conducted another study focusing on the therapeutic potential of MSC-NPs in MS, specifically examining the effects of MSC-NPs on microglial activation, a critical component in MS pathophysiology, particularly the transition from a proinflammatory to a pro-regenerative state (Harris et al., 2023a).

This study utilized *in vitro* models of microglia, including BV-2 mouse microglia cell lines and human induced pluripotent stem cell (iPSC)-derived microglia, to investigate the interaction between MSC-NPs and microglia. Results showed that microglia treated with MSC-NP-conditioned media exhibited a significant decrease in proinflammatory markers and an increase in pro-regenerative markers, suggesting that MSC-NPs may modulate microglial activation through the release of specific factors. A key discovery was the role of transforming growth factor-beta (TGF-β) in mediating the immunomodulatory activities of MSC-NPs toward microglia. The study found that inhibiting TGF-β signaling prevented MSC-NP-conditioned media from inducing beneficial effects on microglia, highlighting TGF-β3 as a mediator of MSC-NPs' therapeutic impact.

In a parallel investigation in 2023, another study by Sadiq's team aimed to clarify the differences in gene expression between MSCs derived from donors with secondary progressive MS (SPMS) and primary progressive MS (PPMS), and corresponding healthy donors, revealed similarities among all groups. Using RNA sequencing analysis, they identified only a few differentially expressed genes between MS and control groups, suggesting a fundamental similarity in MSC gene expression regardless of the MS disease state (Harris et al., 2023b).

Further studies focused on the transformation of MSCs into MSC-NPs, which showed a significant change in the transcriptomic signature. The gene expression profile of MSC-NPs distinctly differed from their MSC counterparts, with numerous genes being up- or down-regulated. This shift indicated a clear cellular identity for MSC-NPs, characterized by downregulation of cell cycle genes and upregulation of genes related to nervous system development, cell signaling, and extracellular matrix organization. Indeed, MSC-NPs exhibited reduced proliferative capacity, associated with the suppression of cell cycle-related genes. However, the upregulation of neural genes underscored the neural progenitor-like nature of MSC-NPs, potentially beneficial in regenerative medicine in MS. An interesting finding was the upregulation of genes involved in the humoral immune response and complement activation in MSC-NPs, suggesting a potential immunomodulatory role that may be relevant to MS treatment. The correlation between C3 secretion and gene expression in MSC-NPs offers insights into possible mechanisms through which MSC-NPs might exert therapeutic effects, particularly in fostering a favorable environment for tissue repair and potentially modulating the immune response in MS.

This comprehensive investigation not only confirms the high degree of similarity among MSCs from MS and non-MS donors but also highlights the significant changes that occur during differentiation into MSC-NPs. The unique transcriptomic profile and functional properties of MSC-NPs offer a distinct therapeutic potential, possibly through the modulation of pathways involved in neural development, extracellular matrix remodeling, and immune response regulation.

## Therapeutic potential of MSC-NPs in MS patients

After conducting various studies at the experimental level and analyzing human samples that underscored the potential of this MSCs therapeutic strategy, Sadiq's team initiated a pilot clinical trial to delve deeper into the mechanistic pathways underpinning the positive effects of this approach. They conducted a study to assess the therapeutic impact of directly delivering MSC-NPs via intrathecal injection in MS patients (Harris et al., 2016). The MSC-NPs used in this study were derived from the bone marrow MSCs of the patients themselves, cultured under specific conditions to promote their differentiation into MSC-NPs. This exploratory pilot feasibility study included 6 patients with progressive MS. The study aimed to evaluate the safety, feasibility, and dose-finding of intrathecal MSC-NP therapy. Each subject was given 2–5 intrathecal doses of autologous MSC-NPs in a dose-escalation fashion. The study reported no adverse concerns. Notably, measurable clinical improvements were observed in four out of six patients. These findings not only confirm the initial tolerability of autologous MSC-NP treatment for MS but also lay the groundwork for subsequent clinical trials.

Building on the encouraging results concerning the risk profile from the initial study, a Phase I clinical trial was initiated to delve deeper into the tolerability and risk assessment of intrathecal delivery of autologous MSC-NPs in progressive MS (Harris et al., 2018). This open-label, single-arm trial involved 20 patients who received three intrathecal injections of autologous MSC-NPs at 3 months intervals. Participants were clinically diagnosed with either SPMS or PPMS and had significant disability but stable disease status in the year prior to the trial. A comprehensive pre-treatment and post-treatment assessment was conducted using various methodologies: EDSS scores, MRI scans, muscle strength grading, urodynamic testing, and evaluations of walking ability. This comprehensive approach was employed to systematically monitor adverse effects and discern any indicative trends of benefit.

Overall, MSC-NP treatment exhibited a risk-free profile throughout the trial, with no significant adverse events or complications attributable to the treatment. The majority of reported adverse events were mild to moderate, such as transient headaches and fever following treatment, which either resolved with basic intervention or required no intervention at all. MRI scans did not detect new T2 lesions or significant changes in disease burden following treatment, further supporting the treatment's safety. Clinically, the study observed improvement in a subset of patients, particularly in EDSS scores, muscle strength, walking ability, and bladder function. Notably, 75% of the patients demonstrated neurological improvement in one of the evaluated domains after treatment, with muscle strength improvements most frequently observed in the lower limbs, aligning with the intrathecal mode of MSC-NP delivery.

The long-term follow-up, conducted 2 years after initiating treatment, did not report any serious adverse events related to the treatment, thus affirming the safety of intrathecal MSC-NP administration (Harris et al., 2021). The adverse events recorded were of a minor nature, such as headaches, which resolved spontaneously without any intervention, suggesting a well-tolerated therapy. Moreover, brain MRI scans revealed no adverse changes throughout the study, further corroborating the treatment's reliability.

Regarding efficacy, continued gains in neurological function were noted in a subset of patients 2 years post-treatment. Of the eight patients who showed improvement at the 6-month mark, seven continued to exhibit positive outcomes, with several ambulatory patients significantly improving their walking speed. The sustained remission of disability underscores the potential of MSC-NP therapy to provide long-term benefits to progressive MS patients, although the extent of improvement varied among subjects. Biomarker analysis via CSF, conducted during the study, revealed the mechanistic actions of the treatment. Significant decreases in the proinflammatory chemokine CCL2 and increases in TGF-β2 post-treatment suggested anti-inflammatory and potentially regenerative actions of the MSC-NPs. Additionally, differential changes in other biomarkers, including HGF, CXCL12, IL-8, and NFL, among responders and non-responders, illuminated the treatment's impact on the CNS environment and its correlation with clinical outcomes.

**Figure 1:**
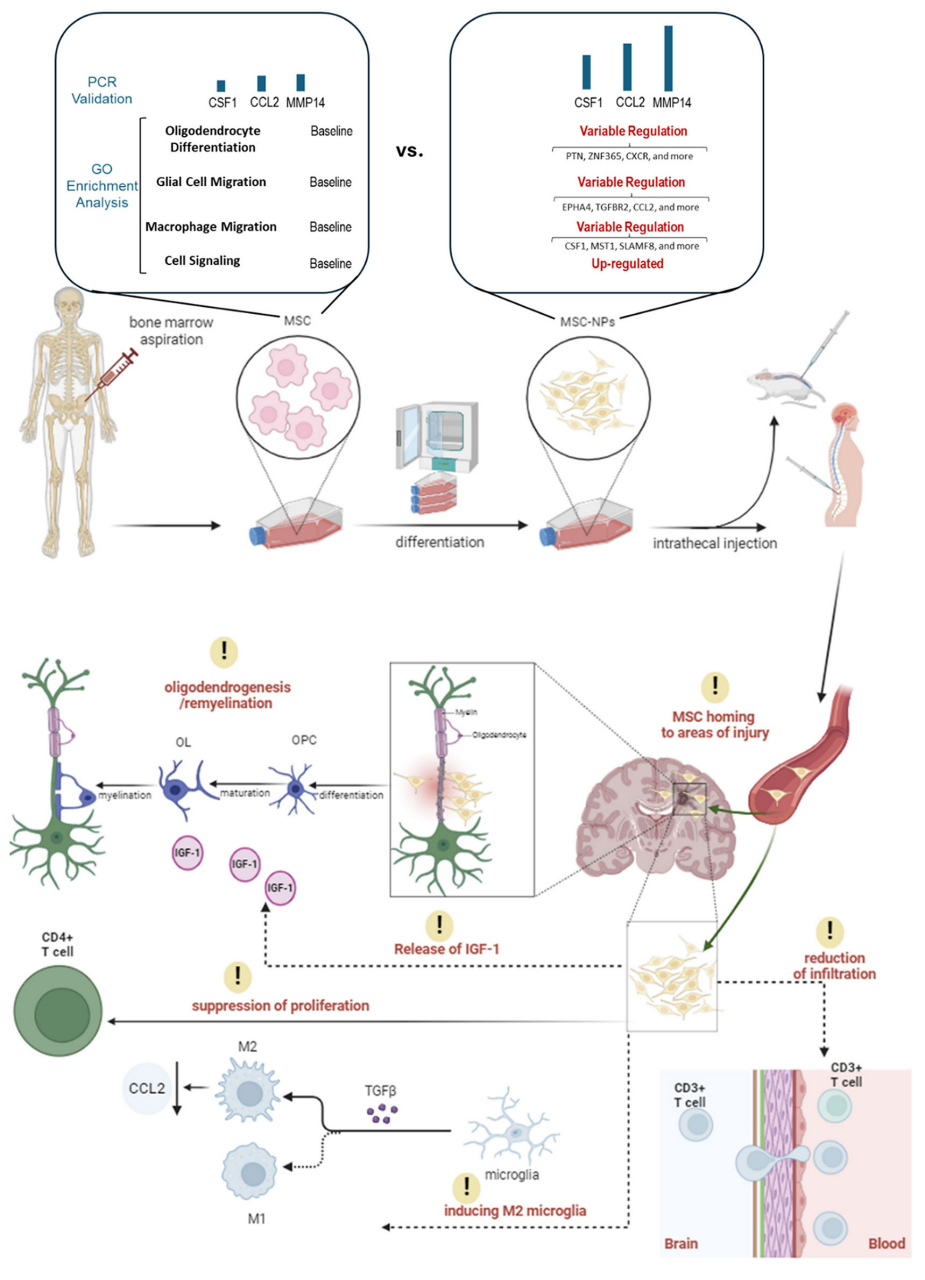
An illustration of MSC-NP therapy in MS patients. This figure presents the therapeutic application of MSC-NPs in the treatment of MS. It begins with the aspiration of MSCs from the bone marrow, which are then induced to differentiate into MSC-NPs. Significant changes in gene expression related to cell migration, signaling, and oligodendrocyte differentiation are observed during this process. Once differentiated, the MSC-NPs are intrathecally administered, directly delivering the cells to the CNS. Following administration, the MSC-NPs exhibit a targeted homing ability, seeking out and accumulating in areas of CNS injury. Their presence leads to a reduction in CD3+ T cell infiltration in the brain, which denotes a significant immunomodulatory effect and suggests a decrease in inflammatory responses. Additionally, the MSC-NPs contribute to oligodendrogenesis and remyelination, potentially facilitated by the secretion of IGF-1, aiding in the repair or support of the myelin sheath. Another aspect of their therapeutic action is the suppression of CD4+ T cell proliferation, further demonstrating their immunomodulatory capabilities. Moreover, the MSC-NPs appear to induce a phenotypic switch in microglia from a pro-inflammatory M1 state to a pro-regenerative M2 state, mediated by the secretion of TGF-β.

Building on the positive results of the Phase I trial, a Phase II trial was initiated in 2018 to further evaluate the efficacy and safety of intrathecal administration of MSC-NPs in patients with progressive MS. Figure 1 simplifies the presentation of MSC-NP therapy in MS and Table 2 summarizes the current clinical trials on MSC and MSC-NPs. Additionally, a concise protocol for the isolation, evaluation, and preparation of MSC-NPs for transplantation in MS patients and the EAE mouse model is detailed in Table 3.

**Table 2:** Clinical studies on the role of MSC and MSC-NP in MS patients.

**References**	**Type of MSC/NP**	**Patient group**	**Route of administration**	**Main findings**	**Adverse effects**
Yamout et al. (2010)	MSC	10 progressive MS patients	Intrathecal	Improvement in EDSS scores; New lesions in some patients	Headaches, other minor symptoms
Karussis et al. (2010)	Autologous MSC	15 MS patients	Intrathecal	Rise in CD4+ CD25+ regulatory T cells. Reduced lymphocyte proliferation. Higher expression of dendritic cell markers post-transplant.	Mild transient symptoms
Bonab et al. (2012)	Autologous MSC	25 Progressive MS	Intrathecal	Three participants withdrew, 4 patients improved, 6 worsened, 12 remained stable. MRI: 15 patients stable; 6 developed new T2/gadolinium-enhanced lesions.	Transient adverse events post-injection: low-grade fever, nausea/vomiting, leg weakness, headache.
Cohen et al. (2018).	Autologous MSC	26 Progressive MS	Intravenous	0.25% had gadolinium-enhancing lesions on MRI. Average MSC dose: 1.9 × 10^∧^6 cells/kg (range 1.3–2.0), 1–3 cell passages. Post-thaw cell viability consistently ≥95%. Infusions were well-tolerated	Without severe or serious adverse events
Mohajeri et al. (2011)	MSC	7 relapsing-remitting MS	Intrathecal	Post-treatment FoxP3 levels were significantly higher at 6 months in almost all subjects. Increased FoxP3 expression correlated with clinical stability.	Mild adverse effects
Petrou et al. (2021)	MSC	24 Progressive MS	Intrathecal (IT) or Intravenous (IV)	22/24 patients were stable or improved at the last follow-up visit; 10 patients showed a reduction in EDSS below baseline levels, with most improvements in those receiving >2 treatments; EDSS score decreased; a transient increase in CD4+CD25+FoxP3+ cells and a reduction in lymphocyte proliferation.	Headaches, minor symptoms
Petrou et al. (2022)	MSC	48 Progressive MS	Intrathecal (IT) or Intravenous (IV)	NFL levels in CSF significantly decreased 6 months after MSC-IT treatment; 9/15 patients in the MSC-IT group saw over a 50% reduction in NFL; Smaller reductions in MSC-IV group (5/15 patients) and an increase in the placebo group (1/15); Decrease in CXCL13 levels after MSC-IT treatment was not statistically significant.	Not reported
Meng et al. (2018)	UCMSC	2 MS patients	Intravenous	Symptoms amelioration: No clinical attacks were reported during the transplantation period; MRI results indicated a reduced number of foci. EDSS scores decreased; Significant decrease in mRNA expression of CD86, IL-2, CTLA-4, and HLADRB1 in peripheral blood	Some patients experienced adverse reactions post-transplantation, which were minor and transient, resolving without intervention
Planchon et al. (2018)	MSC	25 MS patients	Intravenous	Target MSC dose was achieved within 16–62 days, requiring 2–3 cell passages. Growth rate and culture success were not linked to demographic or MS disease characteristics. Cytogenetic studies revealed chromosomal changes in one control (4.3%) after extended culture time.	Mild and transient adverse reactions
Harris et al. (2018)	MSC-NP	6 Progressive MS	Intrathecal	Escalating doses were administered. Patients were followed for an average of 7.4 years post-initial injection. Treatment regimen was well-tolerated. Four out of six patients demonstrated measurable clinical improvement	No safety concerns or serious adverse events were noted.
Harris et al. (2021)	MSC-NP	20 Progressive MS	Intrathecal	Improved median EDSS, indicating potential efficacy. Positive outcomes more common in SPMS patients and those with EDSS ≤ 6.5. 70% of subjects showed improved muscle strength; 50% had better bladder function post-treatment.	Minor adverse events: transient fever and mild headaches, resolving in < 24 h.

**Table 3:** A brief protocol for MSC-NP isolation, evaluation, and preparation for transplantation in MS patients and EAE mouse model, according to the protocol of Sadiq's team.

**Aspect**	**MS patients**	**EAE mouse model**
Source and isolation	Primary MSCs isolated from bone marrow aspirates using plastic adherence and Ficoll gradient centrifugation. MSCs expanded in MSCGM containing 10% autologous serum, serially passaged at 80% confluency, and cryopreserved after 2 and 3 passages.	MSCs derived from adult C57Bl/6 mice. Femurs and tibias dissected and flushed with 2% FBS in PBS to collect bone marrow cells.
Expansion conditions	For each treatment, thawed MSCs expanded for two to three more passages, ranging from 7 to 54 days.	Bone marrow cells plated in proliferation medium at 1 × 10^6^ cells/cm^2^. Non-adherent cells removed every 3–4 days. Cells passaged at 70% confluence with 0.25% trypsin up to 20 passages. MSCs used between P8 and P17, free of hematopoietic cell contamination (CD45–/CD11b–).
Differentiation to MSC-NPs	Cultured in low-adherence flasks in NPMM with 20 ng/mL of EGF and bFGF. Neurospheres formed and cultured for 7 to 24 days, media changed every 2–3 days.	MSCs cultured in low-adherence flasks in NPMM containing NSF-1, EGF, and bFGF for 21 days. Neurospheres formed after 2 days.
Preparation for injection	MSC-NPs collected, washed, counted, resuspended in sterile saline, viability assessed by trypan blue.	Neurospheres converted to single cell suspensions using TrypLE, centrifuged, and triturated with a sterile glass pipette. Cells washed in PBS, viability confirmed by trypan blue (>80% viable).
Validation of MSC-NP purity	• Tested for MSC morphology and surface marker expression (CD105+/CD73+/CD90+/CD45–/CD34–/CD14–/CD19–/HLA-DR–).	• Flow cytometry for CD45, Sca-1, CD9, CD44, and NF-M.
	• *In vitro* osteogenic and adipogenic potential assess using alizarin red and oil red O staining, respectively.	• qPCR for Nestin, NF-M, GFAP, Sca-1, SMA.
	• Cytogenetic analysis for chromosomal stability.	• Adipogenic and osteogenic differentiation tests to confirm reduced mesodermal differentiation capacity.
	• PCR tests for mycoplasma (Sigma) and eubacteria (Minerva Biolabs; Sterility Testing)	
	• MSC-NP Identity Confirmation: Neurosphere morphology and real-time PCR assessing ≥2-fold upregulation of neural markers (nestin, neurofilament M, GFAP, CXCR4) and ≥2-fold downregulation of MSC markers (SMA, CD90).	

Despite the promising therapeutic potential of MSC and MSC- MSC-NP therapies in treating MS, several limitations warrant consideration. The long-term efficacy and safety of these therapies remain uncertain, necessitating further large-scale controlled trials to establish robust clinical evidence. Immunogenicity and the risk of alloimmunization present notable concerns, particularly with allogeneic MSCs, potentially leading to reduced therapeutic effectiveness and patient safety. Moreover, regulatory and ethical considerations are crucial as these therapies advance toward broader clinical application, requiring careful navigation to ensure compliance and ethical integrity. Addressing these limitations is essential for the advancement of MSC and MSC-NP therapies from experimental stages to reliable clinical treatments.

Moreover, the scalability of MSC-NP production for widespread clinical application poses significant challenges. Standardizing the preparation protocols of MSC-NPs is critical to ensure consistency and reproducibility across different laboratories and clinical settings. Variations in cell source, culture conditions, and handling techniques can lead to inconsistencies in the quality and therapeutic efficacy of MSC-NPs. Establishing robust, standardized protocols is essential to overcome these variations, facilitating regulatory approval and ensuring that treatment outcomes are predictable and reliable across various healthcare settings. Additionally, scaling up production while maintaining stringent quality control poses logistical and technical challenges that require innovative solutions to meet the demands of large-scale clinical applications.

We note that the creation of MSC-NP banks is a major advancement in regenerative medicine, providing a potential approach for improving the availability and standardization of cell therapies for MS and other neurodegenerative diseases. Thus, the conception of the MSC-NP biorepository with characterized cells will allow for the faster start of the treatment, decrease the costs of the personalized cell manufacturing, and guarantee the uniform quality of the cellular products. Also, MSC-NP banks could help different research and clinical trials by offering uniform and easily obtainable cell lines. However, this is only possible if there is proper establishment of cryopreservation methods that ensure that the cells are functional even after thawing. It also calls for the development of clear policies and standards on how these cell products should be obtained, stored, and used in order to achieve positive results in the various fields of application.

To sum up, the therapeutic capabilities of MSC-NPs in the treatment of MS serve as a promising development in regenerative medicine. Over the past decade, MSC-NPs have emerged as potentially effective therapeutic agents for addressing both the autoimmune and neurodegenerative aspects of MS, with evidence of safety, tolerability, and efficacy in promoting neurological improvements in progressive MS patients following successful preclinical studies that have led to phase I and II clinical trials. The dual action, the capability of MSC-NPs is pointed to by these results, proposing an attractive therapeutic approach that could greatly change the MS treatment field, however it still need for further studies to completely reveal mechanisms of action and for enhancing the therapeutic efficiency accordingly.
